# Patient stratification in clinical glaucoma trials using the individual tear proteome

**DOI:** 10.1038/s41598-018-30369-x

**Published:** 2018-08-13

**Authors:** Janika Nättinen, Antti Jylhä, Ulla Aapola, Minna Parkkari, Alexandra Mikhailova, Roger W. Beuerman, Hannu Uusitalo

**Affiliations:** 1 SILK, Department of Ophthalmology, Faculty of Medicine and Life Sciences and BioMediTech, University of Tampere, Tampere, Finland; 2 Singapore Eye Research Institute, Singapore, Singapore; 3 Duke-NUS Medical School Ophthalmology and Visual Sciences Academic Clinical Program, Singapore, Singapore; 4 Tays Eye Centre, Tampere University Hospital, Tampere, Finland

## Abstract

Glaucoma patients are prone to concomitant ocular surface diseases; however, switching from preserved to preservative-free medication can often alleviate these symptoms. The objective of this study was to examine how the adverse effects and tear proteome change for glaucoma patients (n = 28) during a 12-month drug switch from preserved latanoprost (Xalatan) to preservative-free tafluprost (Taflotan). We hypothesized that patient stratification could help identify novel recovery patterns in both tear proteomics and clinical data. In order to accomplish patient stratification, we implemented sequential window acquisition of all theoretical mass spectrometry (SWATH-MS) as a tool for quantitative analysis of individual tear protein profiles. During each visit (baseline and four follow-up visits), the patients’ tears were sampled and the state of their ocular surface was evaluated clinically. Altogether 785 proteins were quantified from each tear sample using SWATH strategy and as these protein expression levels were compared between baseline and 12-month follow-up, three distinct patient groups were identified. We evaluated how these patient groups differed in their protein expression levels at baseline and discovered that the patients with increased levels of pro-inflammatory proteins and decreased levels of protective proteins benefitted most from the medication switch.

## Introduction

Glaucoma is a collection of diseases, which can all ultimately result in degeneration of the optic nerve and blindness^1^. In order to halt the glaucomatous changes in the eye, glaucoma treatments attempt to lower the elevated intraocular pressure (IOP), one of the most frequent characteristics of glaucoma, via topical and oral drugs, laser procedures or surgery. Topical treatment is currently the most common glaucoma management method. However, a number of previous studies have shown that prolonged use of topical glaucoma medication may induce symptoms and signs of ocular surface disease, chronic inflammation and other anterior segment diseases^2–4^. The exact cause of the adverse effects are debated, but they could be caused by the active compounds in the eye drops or by the solution preservative such as benzalkonium chloride (BAK) – the most well-known and commonly used preservative in topical glaucoma medication^3,5,6^. Clinical evidence suggests that patients suffering from adverse reactions whilst using preserved topical treatments generally benefit from a switch to preservative-free eye drops: their adverse reactions diminish without compromising the control of IOP^7–12^.

Tear proteome has shown its potential in identifying biomarkers for inflammatory responses associated with glaucoma medication; Wong *et al*.^13^ studied the differences in tear proteomics between glaucoma and control patients and Funke *et al*.^14^ examined the common proteomic changes after medication switch during a 6-month follow-up study with pooled patient samples. Proteomic biomarkers have also been successfully utilized to monitor other eye diseases including dry eye, diabetic retinopathy and age-related macular degeneration^15–22^. To achieve the necessary precision, stratified patients within subgroups must have individual analysis. This is now feasible with label-free mass spectrometry methods, such as sequential window acquisition of all theoretical mass spectra (SWATH-MS), which enable studies of proteomic profiles of each individual patient even in large clinical trials^15,23^. Benefits of label-free MS include analysis of complex comparisons between clinical findings and the individual tear proteome^24^. This approach could become more widely used, if successful bioinformatic methods are developed. For example, we were able to examine why patients react in different ways to the same therapy and study the underlying biological explanation. Focusing on patient stratification is the next natural step in medical research and is expected to become more popular in the future as the need for precision medicine rises^25,26^.

The aim of this study was to evaluate tear protein profiles of individual patients with ongoing glaucoma therapy including BAK-preserved prostaglandin analogue, and proteomic changes after switching to preservative-free medication for a 12-month follow-up period. Our hypotheses were that the patients affected by the switch would also have noticeable changes in their tear protein profiles and that patient stratification could help identify novel recovery patterns in both proteomics and clinical data. Tear proteomics studies on glaucoma have been published previously^13,14^; however, our study is the first to our knowledge to use a precision medicine approach as well as SWATH and to stratify the glaucoma patients into groups based on their individual proteomic responses to medication switch.

## Results

### Study population characteristics and clinical results

The study population consisted of 28 patients (7 men and 21 women). Twenty-five patients were diagnosed with primary open-angle glaucoma and 3 with capsular glaucoma. The mean age of the patients in the beginning of the study was 67.4 years (95% CI: 64.5–70.3). The patients had been on preserved latanoprost treatment for 7.7 years on average (95% CI: 6.1–9.2).

Majority of the clinical signs and symptoms steadily improved throughout the 12 months after switch (Tables 1 and 2). More specifically, the conjunctival redness and lid redness decreased while (fluorescein tear break-up time in seconds) FTBUT and Schirmer’s test values increased. Although the corneal and conjunctival staining scores did not change considerably, the overall means suggest that the scores decreased. In addition, all symptoms experienced by the patients improved after the switch, although some of these improvements were not statistically significant.

**Table 1:** Changes in clinical signs between baseline and the visits after medication switch.

Clinical sign		Baseline values	Change from baseline			
			1.5 months	3 months	6 months	12 months
Conjunctival redness	Mean	1.750	−0.286	−0.607	−0.75	−0.964
	95% CI	[1.459, 2.041]	[−0.541, −0.03]	[−0.873, −0.341]	[−1.022, −0.478]	[−1.188, −0.741]
	P^a^		0.04*	<0.001***	<0.001***	<0.001***
Lid redness	Mean	0.857	−0.357	−0.214	−0.464	−0.519
	95% CI	[0.605, 1.109]	[−0.574, −0.14]	[−0.48, 0.052]	[−0.733, −0.196]	[−0.836, −0.201]
	P^a^		0.004**	0.12	0.003**	0.005**
Fluorescein tear break-up time	Mean	5.393	1.786	2.179	3.214	4.444
	95% CI	[4.284, 6.501]	[0.453, 3.118]	[0.735, 3.622]	[1.531, 4.897]	[2.184, 6.705]
	P^b^		0.01*	0.005**	<0.001***	<0.001***
Corneal staining	Mean	0.607	−0.429	−0.357	−0.286	−0.393
	95% CI	[0.252, 0.963]	[−0.785, −0.072]	[−0.741, 0.026]	[−0.732, 0.16]	[−0.792, 0.007]
	P^a^		0.02*	0.08	0.25	0.06
Conjunctival staining (nasal)	Mean	1.393	−0.25	−0.286	−0.393	−0.179
	95% CI	[1.070, 1.715]	[−0.654, 0.154]	[−0.633, 0.062]	[−0.806, 0.02]	[−0.614, 0.257]
	P^a^		0.31	0.12	0.07	0.53
Conjunctival staining (temporal)	Mean	1.571	−0.321	−0.393	−0.464	−0.357
	95% CI	[1.265, 1.878]	[−0.656, 0.013]	[−0.819, 0.034]	[−0.852, −0.077]	[−0.755, 0.041]
	P^a^		0.07	0.07	0.02*	0.08
Schirmer’s test	Mean	12.000	4.214	4.964	4.786	5.429
	95% CI	[8.511, 15.489]	[1.987, 6.441]	[1.349, 8.579]	[1.288, 8.283]	[2.622, 8.236]
	P^b^		<0.001***	0.009**	0.009**	<0.001***

CI, confidence interval.

*P < 0.05; **P < 0.01; ***P < 0.001; ^a^2-group Wilcoxon Signed Rank Test; ^b^Paired t-test.

**Table 2:** Changes in clinical symptoms between baseline and the visits after medication switch.

Clinical symptom		Baseline values	Change from baseline			
			1.5 months	3 months	6 months	12 months
Irritation, burning, stinging	Mean	1.143	−0.071	−0.179	−0.143	−0.143
	95% CI	[0.629, 1.657]	[−0.738, 0.595]	[−0.855, 0.498]	[−0.849, 0.563]	[−0.737, 0.451]
	P^a^		0.73	0.65	0.61	0.66
Itching	Mean	1.821	−0.643	−0.821	−0.643	−0.75
	95% CI	[1.361, 2.282]	[−1.14, −0.145]	[−1.339, −0.304]	[−1.162, −0.124]	[−1.284, −0.216]
	P^a^		0.02*	0.007**	0.02*	0.01*
Foreign body sensation	Mean	1.571	−0.786	−1	−0.964	−0.393
	95% CI	[1.061, 2.082]	[−1.328,−0.244]	[−1.615, −0.385]	[−1.497, −0.432]	[−1.055, 0.269]
	P^a^		0.008**	0.009**	0.003**	0.30
Tearing	Mean	1.893	−0.929	−1.036	−1.393	−1
	95% CI	[1.321, 2.464]	[−1.466, −0.391]	[−1.673, −0.398]	[−1.934, −0.851]	[−1.517, −0.483]
	P^a^		0.004**	0.009**	<0.001***	0.002**
Dry eye sensation	Mean	2.071	−0.357	−0.536	−0.679	−0.607
	95% CI	[1.555, 2.588]	[−0.855, 0.14]	[−0.951, −0.12]	[−1.247, −0.11]	[−1.294, 0.08]
	P^a^		0.16	0.03*	0.03*	0.09

CI, confidence interval.

*P < 0.05; **P < 0.01; ***P < 0.001; ^a^2-group Wilcoxon Signed Rank Test.

### Relative protein expression levels can be used to stratify patients

We identified a total of 25,487 peptides from 270 samples/MS analysis replicates, corresponding to 388,273 identified spectra in an assembly of 1439 protein groups using FDR of 1.0%. Total of 950 proteins with distinctive peptides were included to quantification library and from this library, 785 proteins had distinct peptide sequences with matching spectra to SWATH analysis and were quantified in all samples. The proteomic data exhibited good quality and reliability with p-value < 0.05 in 89% of replicate MS analyses (permutation tests, Spearman’s rank correlation) and mean intraclass correlation coefficient of 0.97.

**Figure 1:**
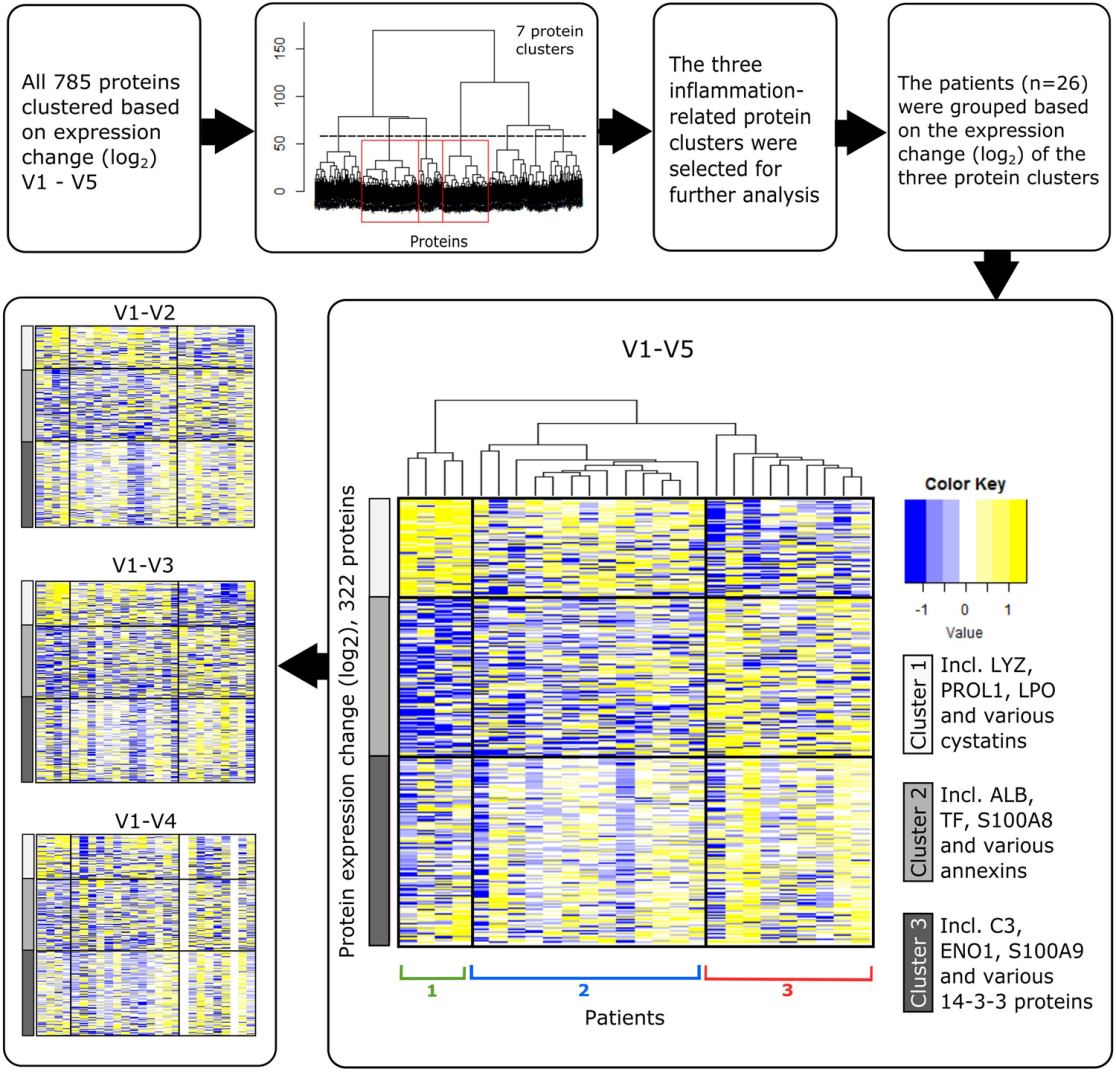
Outline and results from the patient stratification. The top row explains the outline of data processing, protein clustering and patient stratification. The heat maps visualize the change in protein expression between baseline and time points after the medication switch. V refers to visits and focus is on the V1-V5 comparison. Two protein clusters associated with pro-inflammation are indicated in grey rows, and proteins linked to ocular surface protection in white rows. The differences between the patient groups become clearer with time, as is visualized by the additional heat maps (V1-V2, V1-V3 and V1-V4) showing expression differences in comparison to baseline. Two patients were excluded due to missing baseline expression and in addition, two patients had no V4 data. ALB, albumin; C3, complement C3; ENO1, alpha-enolase; LPO, lactoperoxidase; LYZ, lysozyme; PROL1, proline-rich protein 1; TF, serotransferrin.

We wanted to establish how each patient’s protein profile changed during the 12-month treatment period. To achieve this, we first clustered the log_2_ fold changes between the first and final visit, and based on the dendrogram and visual inspection of results we set the cut-off at 7 clusters (Fig. 1). We then conducted pathway analysis with Ingenuity Pathway Analysis (IPA) for all 7 protein clusters and identified three clusters of interest, enriched with inflammatory proteins as shown in Supplementary Table S1. Within these clusters, protein profiles among patients became clearer during the 12-month follow-up (Fig. 1). The first protein cluster included several protective ocular surface biomarkers, such as lysozyme (LYZ), proline-rich protein 1 (PROL1) and various cystatins. Altogether 71 proteins were in this cluster and the top enriched disease and function terms, according to IPA, included “activation of neutrophils” and “chronic inflammatory disorder”. The second cluster included inflammatory biomarkers such as albumin (ALB), serotransferrin (TF), protein S100A8 and annexins, with a total of 116 proteins all displaying similar fold changes. The top enriched terms for this cluster included “inflammation of organ” and “cell death”. The third cluster also included known inflammation biomarkers such as complement C3 (C3), alpha-enolase (ENO1) and protein S100A9. The 135 similarly expressed proteins in this cluster had enrichments relating to cell death, cell movement and inflammation of organ.

Next, it was possible to stratify patients into three groups, based on the changes in filtered proteomic profiles (Fig. 1). The patients in groups 1 and 2 showed somewhat similar improvement based on their proteomic profile: expression of protective proteins increased and pro-inflammatory protein expression decreased. Heat map and clustering analysis further differentiated these groups; protein expression changes were more consistent for group 1 patients, while there was some variation among patients in group 2. The patients in group 3 experienced a decrease in protective proteins’ expression and an increase in expression of pro-inflammatory proteins, suggesting that they were not benefitting from the drug switch.

### Baseline expression levels of several proteins indicate individual differences between the patient groups

**Figure 2:**
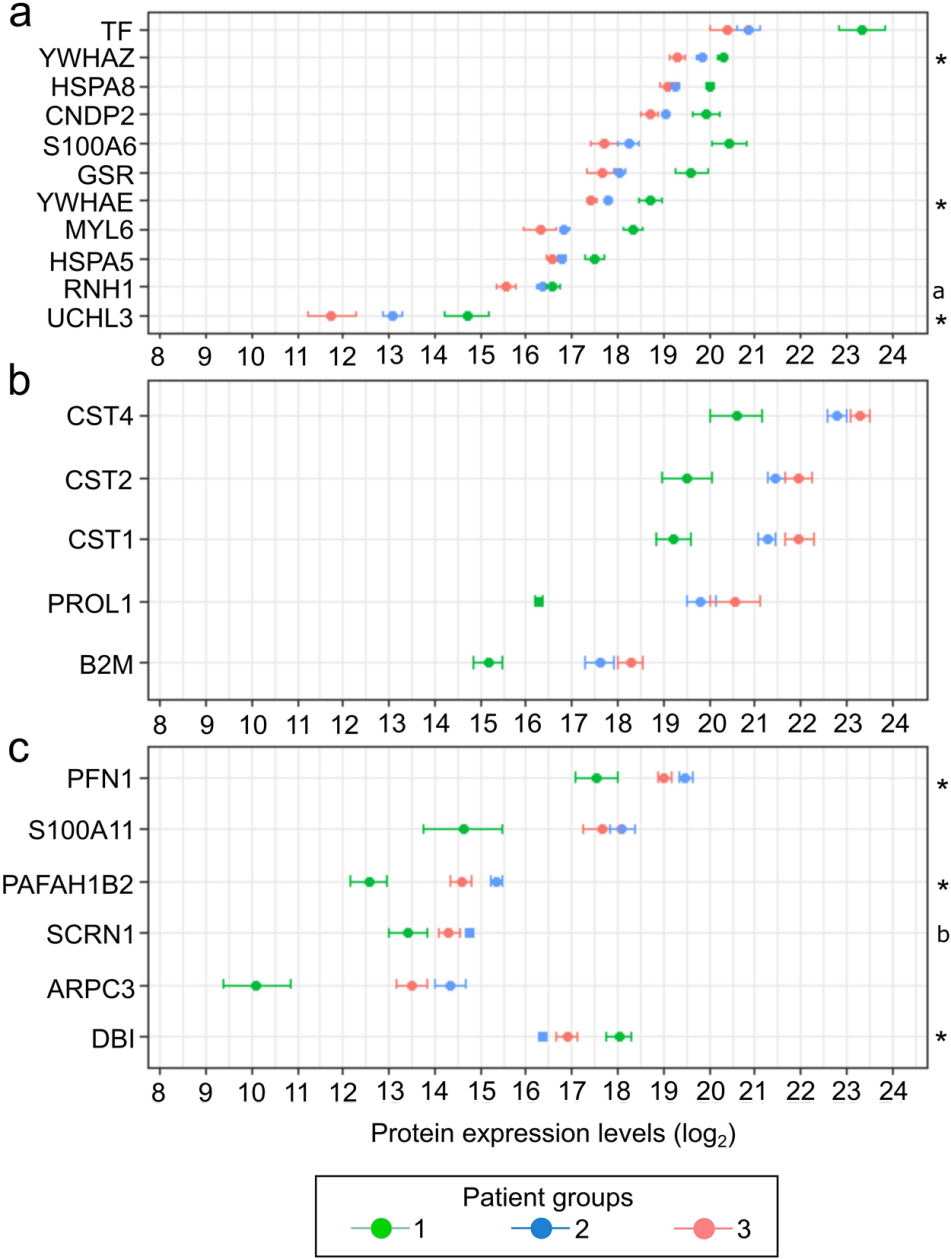
Proteins (y-axis) with differing baseline log_2_ expressions (x-axis) between the patient groups. Protein expression of several pro-inflammatory proteins is highest at baseline for patients in group 1, intermediate in group 2, and lowest in group 3 (**a**). Protein expression levels of various cystatins, proline-rich protein 1 (PROL1), and beta-2-microglobulin (B2M), considered to be beneficial, are lowest for group 1 patients, intermediate in group 2, and highest in group 3 (**b**). A collection of proteins not following the same order as in A and B (**c**). Measures are shown as mean ± s.e.m. and all proteins missing a specification (*, a or b) have a statistical difference between patient groups 1 and 2, and 1 and 3 (Welch’s analysis of variance). *Significant differences between all patient groups; ^a^Patient group 3 differs significantly from other groups; ^b^Patient groups 1 and 2 differ from each other significantly.

Next, we examined if baseline expression levels of individual proteins would differ between the three patient groups. After p-value adjustment, out of 322 clustered proteins, 31 remained statistically significant (p-value < 0.05). We excluded one protein without a gene symbol (immunoglobulin), two proteins with unequal variance (heteroscedasticity, Levene’s test p-value < 0.05) and six proteins with poor peak matches, yielding a total of 22 proteins which differed between the patient groups at the baseline (Fig. 2). Many of the statistically significant proteins were ordered in a similar manner; proteins in Fig. 2a had the highest expression among patients in group 1, then 2 and patients in group 3 had the lowest relative expression levels. This order was reversed for proteins in Fig. 2b, where group 1 patients had the lowest relative expression. The results also included some less consistent results, which could none-the-less provide further, interesting information of the patient groups (Fig. 2c). More detailed statistics results are provided in Supplementary Table S2.

### Protein expression levels correlate with Schirmer’s test and FTBUT values

**Figure 3:**
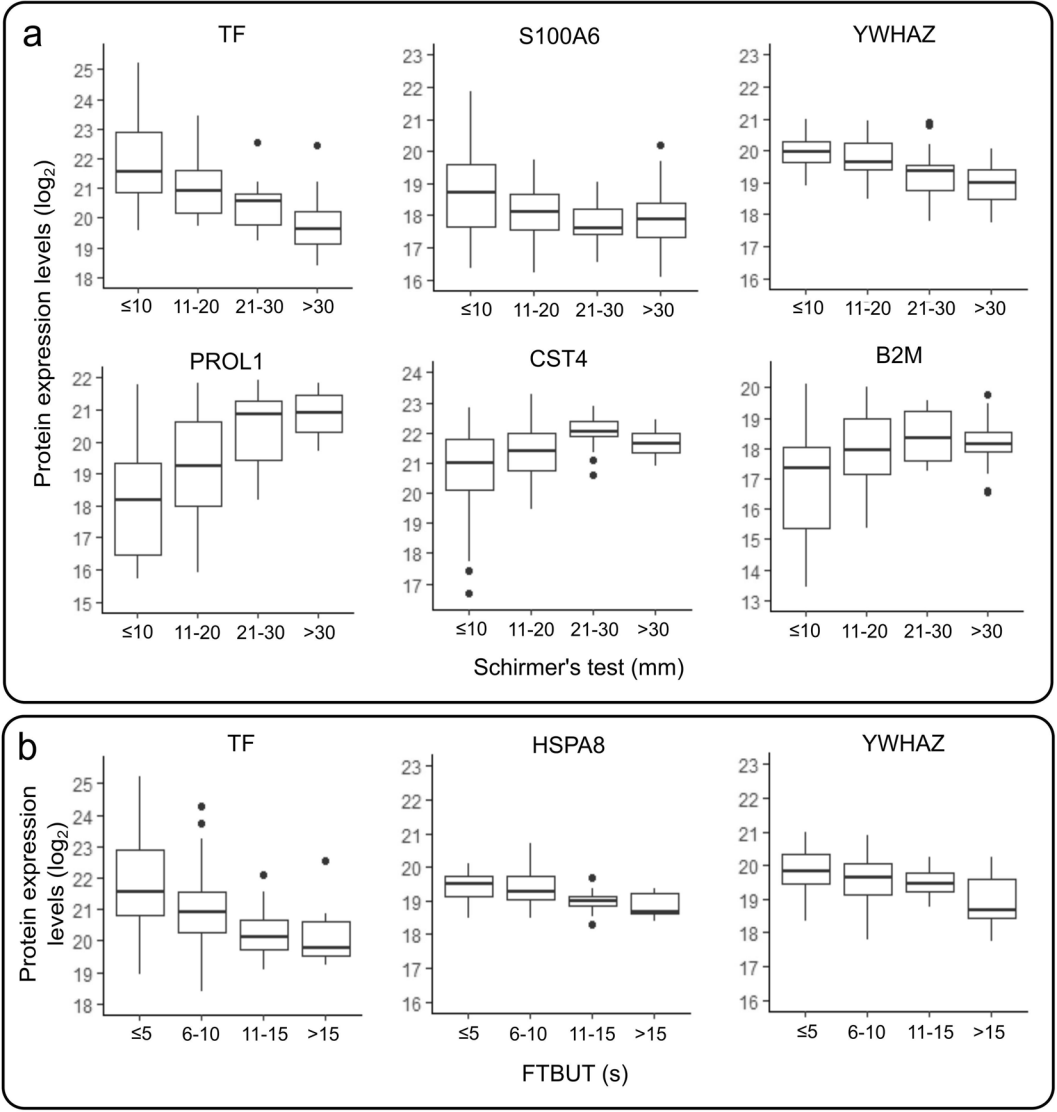
Schirmer’s test and fluorescein tear break-up time (FTBUT) correlate with statistically significant proteins identified in the study. Serotransferrin (TF), S100A6 and 14-3-3 protein zeta/delta (YWHAZ) expression levels correlate negatively with Shirmer’s test, while proline-rich protein 1 (PROL1), cystatin S (CST4) and beta-2-microglobulin (B2M) have a positive correlation (**a**). TF, heat shock cognate 71 kDa protein (HSPA8) and YWHAZ expression levels correlate negatively with FTBUT (**b**). Statistically significant correlations were identified using mixed model regression and the data are shown as boxplots displaying median, 25 and 75 quartiles, 5 and 95 percentiles (error bars). Black dots represent potential outliers.

Next, we wanted to compare clinical results with tear proteomics data and performed mixed effects model analysis. All pro-inflammatory proteins that differed between the patient groups at baseline (Fig. 2a), excluding pro-apoptotic cytosolic non-specific dipeptidase (CNDP2), correlated negatively with Schirmer’s test results (Fig. 3a), and four correlated negatively with FTBUT (Fig. 3b). The cystatins, PROL1 and beta-2-microglobulin (B2M) (Fig. 2b) correlated positively (Fig. 3a) and acyl-CoA-binding protein (DBI) (Fig. 2c) correlated negatively with Schirmer’s test. Full statistical results are available in Supplementary Table S3. To conclude, we observed that Schirmer’s test and FTBUT values correlate negatively with pro-inflammatory proteins and the correlation is positive with protective proteins.

### Patient groups and their differences in clinical signs and symptoms

**Figure 4:**
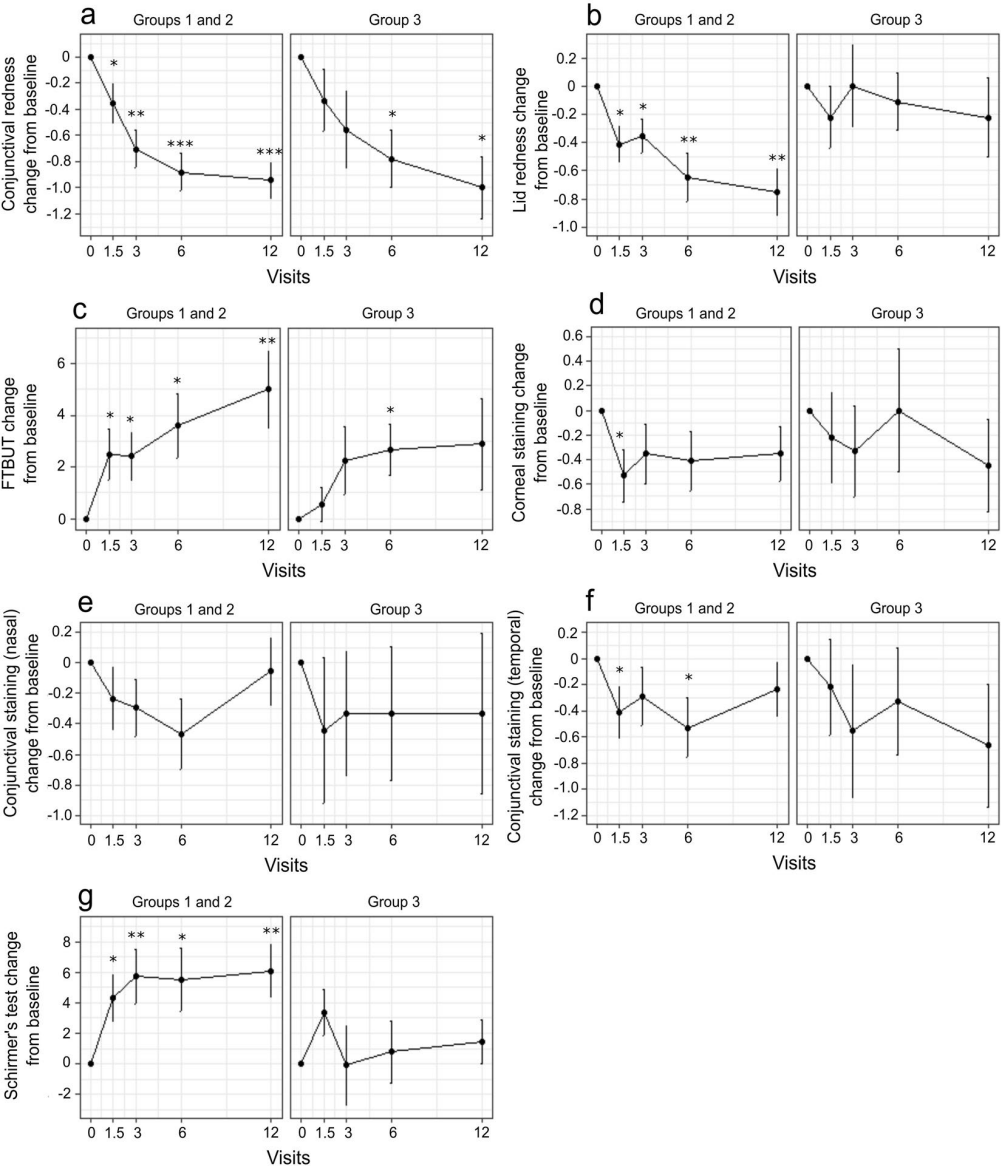
Changes in clinical signs between baseline and the visits after medication switch for groups 1 and 2 (improved patients), and group 3 (unimproved patients). As seen from the visits (x-axis), as time progresses, the mean (±se) change (y-axis) of conjunctival redness (**a**) and lid redness (**b**) decreased for groups 1 and 2 as well as for group 3, yet the changes were only statistically significant for the former. Similarly, fluorescein tear break-up time (FTBUT) (**c**) and Schirmer’s test (**g**) were increased for all patients, however only group 1 and 2 patients had statistically significant improvement. Corneal (**d**) and conjunctival staining (**e**,**f**) were not showing similar signs of improvement, which matches previous results. Measures are shown as mean ± s.e.m change from baseline and continuous signs (**c**,**d**) were analysed with paired t-test and the rest with paired 2-group Wilcoxon signed rank test. *P < 0.05; **P < 0.01; ***P < 0.001.

**Figure 5:**
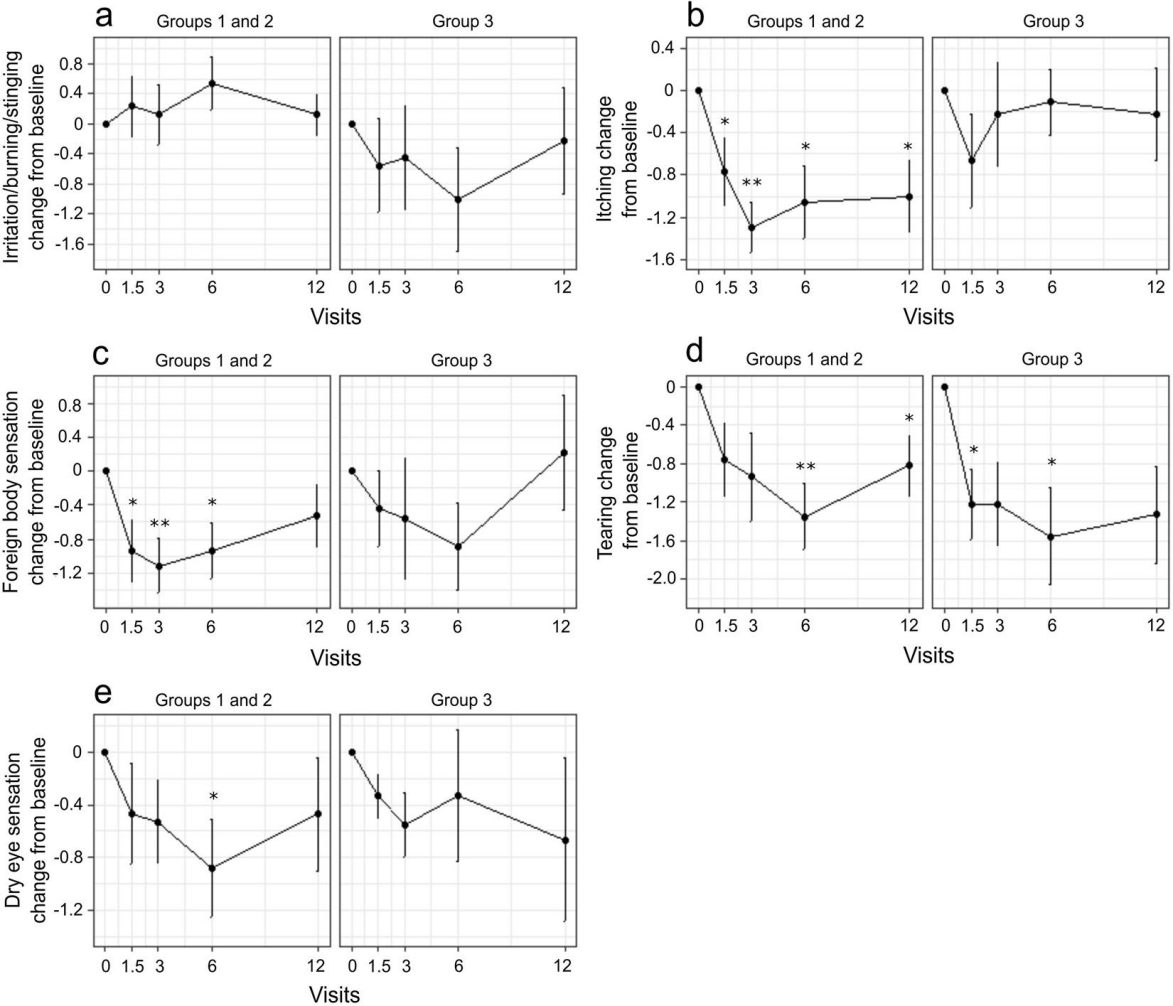
Changes in clinical symptoms between baseline and the visits after medication switch for groups 1 and 2 (improved patients), and group 3 (unimproved patients). Irritation/burning/stinging (**a**) was not changing significantly in any time point (x-axis), while itching (**b**) and foreign body sensation (**c**) were significantly reduced for groups 1 and 2. Some evidence of reduction was also present for tearing (**d**) and dry eye sensation (**e**). Measures are shown as mean ± s.e.m change from baseline and were analysed with paired 2-group Wilcoxon signed rank test. *P < 0.05; **P < 0.01; ***P < 0.001.

Finally, we analysed how the clinical signs and symptoms changed within the identified patient groups. Since patient group 1 had only four patients, we decided to combine groups 1 and 2 and this way, patients groups 1 and 2 together show patients, who appear to benefit from the drug switch and group 3 includes patients who do not. The results for the clinical sign changes showed that while there was beneficial development for both groups 1 and 2 together and group 3, the changes for patient group 3 were not often statistically significant (Fig. 4). For example, Schirmer’s test and FTBUT increased significantly for groups 1 and 2 together but not for group 3. Similarly, conjunctival redness and lid redness decreased throughout the study for groups 1 and 2, but the changes for group 3 were not consistently significant. Same analysis was also performed for the clinical symptoms (Fig. 5). Groups 1 and 2 had more significant decreases in itching and foreign body sensation while irritation/burning/stinging, tearing and dry eye sensation changes were not as consistent with previous findings.

## Discussion

In our study, as with previously published studies, patients experiencing adverse effects from long-term use of BAK-preserved topical glaucoma medication benefitted from a switch to a preservative-free topical treatment according to majority of the clinical signs. However, based on our previous clinical data^7,8^ and the present proteomic data stratification, the level of this improvement varied among patients. In order to examine differences between patients on proteome level, we stratified patients based on expression changes between baseline and final visit (V1-V5) and identified differences in proteins connected to inflammation. Our results showed that the patients had different patterns of protein expression, which became more consistent and clear with time, forming three patient groups towards the end of the study: greatly improved (group 1), moderately improved (group 2) and unimproved (group 3) proteomic profiles.

When comparing baseline protein expression levels of the three patient groups, we identified 22 proteins that were differentially expressed. The most improved patients (group 1) had higher baseline expression levels of several known pro-inflammatory proteins, which were in relation low in expression for the unimproved patients (group 3). These proteins included the 14-3-3 protein epsilon (YWHAE) and 14-3-3 protein zeta/delta (YWHAZ), which belong to the same protein family and YWHAZ has been previously found to be upregulated in the tears of patients using topical anti-glaucoma medication^13^. Similarly heat shock proteins (HSP) HSPA5 and HSPA8, had increased expression levels among most improved patients in our results. These proteins tend to be highly expressed in glaucomatous eyes^27^ and are associated to environmental stress; however, their connections to glaucoma medication have not been examined. Other similarly expressed proteins connected to ocular inflammation were iron transport protein transferrin (TF)^28^, which has been found to be upregulated in glaucoma and in particular with patients using preserved medication^14,29^, and protein S100A6, which is upregulated in dry eye disease^30^. In addition, myosin light polypeptide 6 (MYL6) had expression level similar to the other pro-inflammatory proteins and interestingly, the myosin light chains (MLC) have previously been linked to BAK-related inflammation^31,32^.

The proteins with low expression among the most improved patients (group 1) and higher expression for the unimproved patients (group 3) included several cystatins (cystatins S (CST4), SA (CST2) and SN (CST1)), lacrimal gland secreted PROL1 and B2M. Of these proteins CST4, CST1, B2M and PROL1 have been found to be decreased in dry eye disease^20,33–35^. Based on these “beneficial” proteins, as well as the pro-inflammatory proteins previously described, the patients who experience the greatest improvement are patients with the highest initial expression levels of pro-inflammatory proteins and lowest expression of protective proteins. This suggests that patients with more severe ocular surface condition benefit from the switch the most and that the differences among patients can be discovered using proteomics.

Next, we wanted to combine all the clinical and proteomic information in the light of our results. More specifically, we wanted to see if there were any statistically significant correlations between the proteins of interest and the clinical signs. We observed that low Schirmer’s test and FTBUT values were associated with high expression of pro-inflammatory proteins and low expression of cystatins, PROL1 and B2M. These statistically significant correlations between the protein expression levels and clinical signs further confirmed the roles of the proteins, which we have previously described.

Finally, we compared the patient groups’ clinical sign and symptom development after the switch and noted that the signs and symptoms were significantly improving for many patients in groups 1 and 2, i.e. those who were benefitting from the drug switch based on proteomics data. Patients in group 3 were also moderately improving, but for the majority of signs and symptoms, this was not statistically significant. This suggests that the pro-inflammatory and protective proteins do identify patients benefitting from the switch also based on clinical parameters, such as Schirmer’s test, FTBUT, corneal staining, lid redness, tearing and foreign body sensation.

Our results suggesting there is a subgroup of patients not benefitting from the switch was not surprising, since the growing consensus is that individual response to medications can widely vary and has been also indicated by previous clinical studies^8^. However, with other studies, the sample sizes have been relatively large and it is possible that smaller subgroups get overrepresented in smaller studies such as ours. One hypothesis to explain the varying changes after switch is that these patients could be more sensitive to active compound of the drug, such as prostaglandin analogue, which is also known to cause adverse reactions on ocular surface^36,37^. Alternatively, these unimproved patients could be suffering from other ocular surface conditions, which are unrelated to the BAK-effects. Either way, this topic deserves further examination.

In our current study, the analysis of the protein expression levels was done with respect to the follow-up data of other patients suffering from adverse effects and by analysing the changes after the omission of preservatives. A control population could have helped us make concrete conclusions about up- or downregulation of proteins. However, we have already begun work to identify the normal expression levels of these proteins in population of normal subjects of various ages.

In conclusion, by implementing SWATH method for quantitative analysis of individual tear protein profiles, we discovered that patients react differently when switched to preservative-free glaucoma medication and their condition continues to change for at least 12 months. This study demonstrates that when analysing the proteomic profiles, the patients should be analysed separately as they experience different changes in expression of inflammation-related proteins. In addition, knowledge of the baseline protein expression is crucial in studies focusing on patient stratification. Hence, in order to obtain versatile data needed for patient stratification, mass spectrometry method should be chosen carefully. The overall results of this study suggest that the patients who have the most severe BAK-induced adverse effects benefit most from the switch and that these patients could be detected using tear proteomics. This further suggests that a subgroup of the patients are suffering from some other, BAK-independent, ocular surface-related conditions, and should be treated accordingly to improve the well-being of these patients. We identified several potential biomarkers, such as pro-inflammatory proteins YWHAE and YWHAZ and various beneficial cystatins, which may indicate whether the patient will benefit from a switch to other therapeutic treatments. Proteomic tear fluid biomarkers provide efficient tools for developing precision therapeutic strategies for glaucoma patients and deserve further studies.

## Methods

### Study population

The study was conducted in accordance with the International Conference of Harmonization Good Clinical Practice guidelines and the Declaration of Helsinki. Study was approved by the Ethics Committee at Tampere University Hospital and was registered in EU Clinical Trials Register (EudraCT Number: 2010-021039-14, registration date: 3/28/2010, online: https://www.clinicaltrialsregister.eu/ctr-search/search?query=eudract_number:2010-021039-14). Each patient signed a written informed consent before inclusion in the study.

The patients were assessed during the baseline visit and eligible patients had primary open angle or capsular glaucoma. The included patients had also been receiving preserved latanoprost treatment for 6 months or longer for both eyes and exhibited at least two ocular symptoms or one symptom and one sign of ocular surface irritation/inflammation. Thirty patients were selected for this study based on these inclusion criteria. One of the patients died during the follow-up and one discontinued the study and hence, the final study population was reduced to 28 patients. Although both eyes were examined for each patient, only the right eyes were included in the analysis.

Patients who were excluded from the study had pigmentary or angle-closure glaucoma, IOP higher than 22 mmHg in baseline, corneal abnormalities affecting tonometry, had undergone a recent (within 6 months) ocular surgery including laser procedures, wore contact lenses, or were using artificial tears containing preservatives. In addition, pregnant and nursing women as well as women of childbearing potential without adequate contraception were excluded.

### Study outline

**Figure 6:**
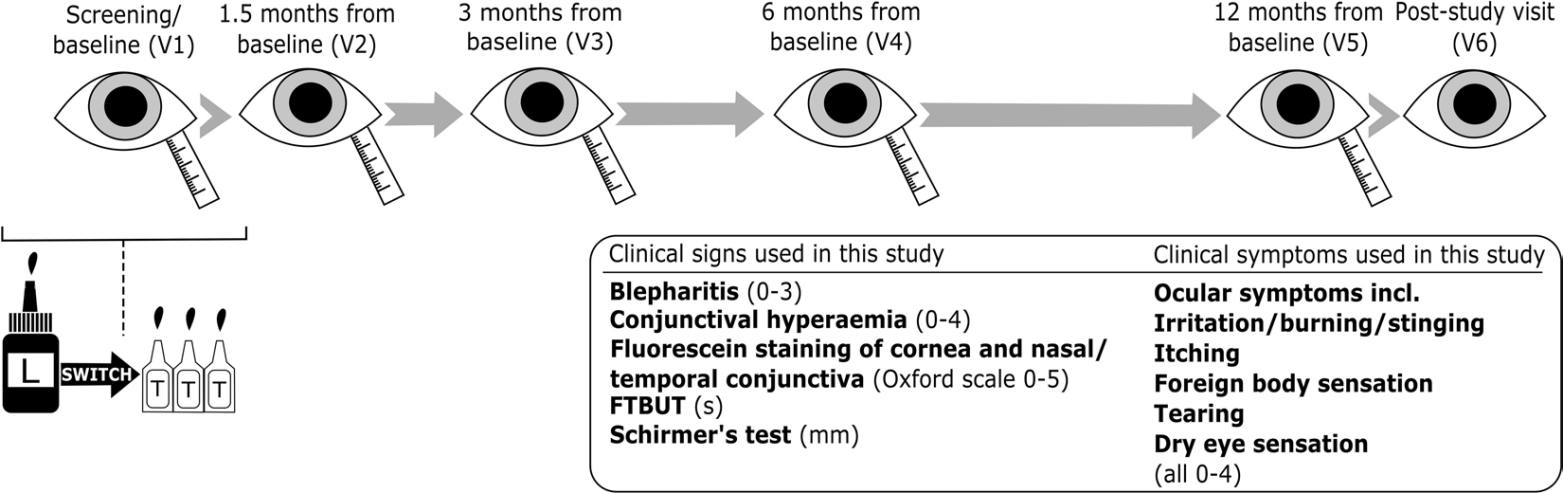
Study outline summary. During the screening/baseline visit (V1), the patients were switched from preserved latanoprost (L) to preservative-free tafluprost (T) in unit dose dispensers. Clinical measurement together with the tear sample collection were performed at visits V1-V5. At the post-study visit (V6), final clinical measures were recorded but tear samples were no longer collected. FTBUT, fluorescein tear break-up time.

The study consisted of 6 visits: screening/baseline visit, visits at 1.5, 3, 6 and 12 months after the baseline, and 1–4 weeks after the 12-month visit (Fig. 6). At baseline visit, the patients were switched from preserved latanoprost (Xalatan, Pfizer Inc., New York, NY, USA) to preservative-free tafluprost (Taflotan, Santen Inc., Osaka, Japan) and their clinical signs and symptoms, and medical history were recorded. The preservative-free eye drops were administered once a day for the duration of the study. The tear fluid samples for proteomics analysis were collected using a Schirmer’s strip and in addition, ocular examinations and procedures were performed during each visit including ocular symptoms (irritation/burning/stinging, itching, foreign body sensation, tearing, dry eye sensation), conjunctival hyperaemia, fluorescein staining of cornea and nasal and temporal conjunctiva, FTBUT, lid redness, and Schirmer’s test. The ocular symptoms were graded between 0 and 4 in the following scaling: none (0), trace (1), mild (2), moderate (3) and severe (4). For the ocular signs, conjunctival hyperaemia was assessed using reference photographs and a similar scale as with ocular symptoms. Fluorescein staining of the cornea and nasal and temporal conjunctiva was measured according to the Oxford grading scale from 0 to 5 and FTBUT was evaluated under a slit lamp microscope (seconds). Lid redness was evaluated as none (0), mild (1), moderate (2) and severe (3) and tear secretion was measured using Schirmer’s test (mm), from which tear proteins were isolated for proteomics analysis. Clinical examinations and sample collections were performed at the same time of the day during each visit. No tear samples were collected during the last visit (V6, ~12.5 months after baseline).

### Tear fluid collection and sample preparation

Patients’ tear fluid samples were collected with Schirmer’s strips without anaesthesia (Tear Touch, Madhu Instruments, New Delhi, India). The strips were inserted under patients’ lower eyelids and removed after 5 min. Tear amounts (mm) were recorded and strips were then stored at −80 °C until proteomic analyses.

For extraction of tear proteins, Schirmer’s strips were first cut into small pieces and solubilized in 50 mM ammonium bicarbonate solution containing Protease Inhibitor Cocktail (Thermo Fisher Scientific Inc., Waltham, MA, USA) for 3 h. Samples were then centrifuged and total protein concentration of the supernatants was measured. Up to 50 µg of protein from each sample was dried in a speed vacuum concentrator. Further information of the methods, including denaturation, alkylation, reduction and tryptic digestion as well as analysis with Eksigent 425 NanoLC coupled with high speed TripleTOF 5600+ mass spectrometer (Ab Sciex, Concord, Canada) can be found from the Supplementary methods and from our previous publication^15^.

### SWATH library creation and peak integration

SWATH library was created with ProteinPilot software version 4.6 (Sciex, Canada). The library was used to analyse MS/MS data and search against the Uniprot reviewed library (Swiss-Prot) for protein identification. Some important settings in the Paragon search algorithm in ProteinPilot were configured as follows. Sample type: identification, Cys-alkylation: IAA, Digestion: Trypsin, Instrument: TripleTOF 5600+, Search effort: thorough ID. False discovery rate (FDR) analysis was performed in the ProteinPilot and FDR < 1% was set for protein identification. The data from all the identification runs from patients were combined as a batch and used for library creation. PeakView software 2.0 with SWATH was used to assign the correct peaks to correct peptides in the library. iRT peptides (Biognosys, Switzerland) were used for retention time calibration with PeakView. 1–12 peptides per protein and 5 transitions per peptide were selected to be used in SWATH quantification. All shared peptides were excluded from analysis. SWATH plug-in FDR Analysis was used to select the proper peptides for use in quantification. All proteins with significant or interesting findings in the data analysis were subjected to manual inspection of peptides. This consisted of checking correct peak selection in the chromatogram (FDR 1%, 99% peptide confidence level), sufficient signal to noise ratio inspection (>7) and chromatogram inspection in relation to library chromatogram. All peptides were eliminated from results processing if manual inspection requirements were not fulfilled.

### Data processing and statistical analysis

Log_2_-transformation and quantile normalization were applied to all quantification results. The majority of the samples had two replicate MS analyses and the variation between them was evaluated by intraclass correlation (ICC package in R) and by permutation tests using Spearman’s rank correlation coefficients. The replicate MS analyses were then combined by taking geometric means.

For the clinical data, two-tailed paired t-test for continuous and paired 2-group Wilcoxon signed rank test for ordinal clinical signs and symptoms were used to evaluate how the clinical signs and symptoms changed during visits. For proteomic data, fold changes (log_2_) between baseline and other visits were analysed using hierarchical clustering (Euclidean distance measure and Ward’s method as the criteria) in order to identify clustered groups of proteins with association to ocular surface complications. The clusters of interest were identified using Ingenuity Pathway Analysis (IPA) and confirmed by identifying well-known biomarkers. The chosen protein clusters were used to group patients based on their proteome changes, again using the same hierarchical clustering method. Welch’s analysis of variance (ANOVA) was used to establish proteins, which could separate these patient groups based on their baseline expression levels alone. Pairwise comparisons were conducted to the statistically significant results, which did not suffer from heteroscedasticity according to Levene’s test for homogeneity of variance (p-value > 0.05). The linear relationship between proteins and the clinical signs was measured either by mixed model regression (lmer function from *lme4* package in R^38^) or cumulative link mixed model (clmm function from *ordinal* package in R^39^) in order to account for the repeated measures from the same patients. The changes in clinical signs and symptoms among patient groups were again analysed conducting paired t-test for continuous and paired 2-group Wilcoxon signed rank test for ordinal variables.

Manual peak checking was implemented for the proteins of interest and as a result, some statistically significant proteins were omitted from the results due to poor peptide matching. Benjamini & Hochberg correction was applied to p-values and only proteins with an adjusted p-value below threshold (alpha = 0.05) were considered unless otherwise stated. All statistical analyses for the proteomics data were performed using R software version 3.2.3 (R Core Team. Foundation for Statistical Computing, Vienna, Austria) and QIAGEN’s IPA (QIAGEN Redwood City, USA).

### Data availability

The data generated and analysed during the current study are not publicly available due to a pending patent application but are available from the corresponding author on reasonable request.

## Electronic supplementary material

Supplementary information
