# Supplementary material for: Patient stratification in clinical glaucoma trials using the individual tear proteome
Source: Sci Rep. 2018 Aug 13;8:12038. doi: 10.1038/s41598-018-30369-x (PMC6089987; doi:10.1038/s41598-018-30369-x)
Supplement: Supplementary file 1 — Supplementary information [file 41598_2018_30369_MOESM1_ESM.pdf]

Patient stratification in clinical glaucoma trials using the individual tear proteome

Janika Nättinen, Antti Jylhä, Ulla Aapola, Minna Parkkari, Alexandra Mikhailova, Roger W Beuerman, Hannu Uusitalo

Supplementary Tables

Supplementary Table S1. IPA pathway analysis of the protein clusters and top 10 diseases or function annotations with the lowest p-values.

	Diseases or Functions Annotation	P[a]
Cluster 1	psoriasis	2.65E-13
	metabolism of protein	1.45E-12
	cell death of tumor cell lines	5.85E-12
	catabolism of protein	1.33E-11
	Rheumatic Disease	2.17E-11
	necrosis	8.45E-11
	amyloidosis	2.90E-10
	aggregation of cells	4.44E-10
	cell death	5.62E-10
	adhesion of blood cells	6.80E-10
Cluster 2 - chosen for further analysis	inflammation of organ	7.23E-12
	psoriasis	1.88E-11
	cell death	1.62E-09
	Dermatitis	8.67E-09
	atopic dermatitis	1.44E-08
	allergy	2.34E-08
	cell death of tumor cells	1.31E-07
	invasion of cells	1.53E-07
	metastasis	1.74E-07
	hypersensitive reaction	3.13E-07
Cluster 3 - chosen for further analysis	cell death	2.09E-16
	cell movement	6.17E-16
	cellular infiltration	2.46E-13
	allergy	1.91E-12
	inflammation of organ	2.34E-12
	neuromuscular disease	2.69E-12
	apoptosis	7.47E-12
	necrosis	8.17E-12
	metabolism of reactive oxygen species	2.30E-11
	chronic fatigue syndrome	3.00E-11
Cluster 4	amyloidosis	2.44E-07
	activation of neutrophils	3.41E-07

- chosen for further analysis	chronic inflammatory disorder	1.21E-06
	aplastic anemia	2.12E-06
	cell death	4.29E-06
	chronic psoriasis	6.61E-06
	Alzheimer's disease	6.91E-06
	chronic inflammatory demyelinating polyradiculoneuropathy	1.08E-05
	rheumatoid arthritis	1.40E-05
	complement component deficiency	1.61E-05
Cluster 5	cell death of tumor cell lines	1.37E-11
	cell death	1.12E-08
	synthesis of reactive oxygen species	2.39E-08
	generation of reactive oxygen species	3.89E-08
	necrosis	1.35E-07
	apoptosis of tumor cell lines	2.51E-07
	advanced non-small-cell lung carcinoma	2.72E-07
	growth of yeast	4.74E-07
	metastasis	6.22E-07
	cell movement	1.96E-06
Cluster 6	Viral Infection	1.62E-07
	synthesis of protein	1.81E-06
	cell death of osteosarcoma cells	2.71E-06
	synthesis of nucleoside diphosphate sugar	1.48E-05
	Baraitser-Winter syndrome	2.06E-05
	movement of duct cancer cell lines	2.06E-05
	metabolism of protein	2.20E-05
	organization of cytoskeleton	2.54E-05
	modification of hydrogen peroxide	2.55E-05
	organization of cytoplasm	3.90E-05
Cluster 7	psoriasis	5.20E-08
	Viral Infection	5.30E-08
	allergy	2.43E-07
	endocytosis	1.14E-06
	atopic dermatitis	1.46E-06
	immediate hypersensitivity	2.21E-06
	Dermatitis	3.03E-06
	metabolism of protein	3.09E-06
	cell death	3.46E-06
	catabolism of protein	6.62E-06

[a]Fisher’s Exact Test (IPA’s default)

Supplementary Table S2. Proteins separating the three patient groups from each other based on their baseline expression values.

Uniprot	Full name	Symbol	Means of groups (log_2)			P between groups[a]		
			C1	C2	C3	C1-C2	C1-C3	C2-C3
P02787	Serotransferrin	TF	23.35	20.84	20.40	<0.001	<0.001	0.34
P01036	Cystatin-S	CST4	20.59	22.79	23.29	<0.001	<0.001	0.10
P11142	Heat shock cognate 71 kDa protein	HSPA8	20.02	19.25	19.08	<0.001	<0.001	0.33
Q96KP4	Cytosolic non-specific dipeptidase	CNDP2	19.93	19.04	18.69	<0.001	<0.001	0.07
P62258	14-3-3 protein epsilon	YWHAE	18.72	17.78	17.43	<0.001	<0.001	0.02
P00390	Glutathione reductase, mitochondrial	GSR	19.60	18.04	17.64	<0.001	<0.001	0.20
P01037	Cystatin-SN	CST1	19.21	21.26	21.97	<0.001	<0.001	0.06
P11021	78 kDa glucose-regulated protein	HSPA5	17.51	16.77	16.58	<0.001	<0.001	0.22
Q99935	Proline-rich protein 1	PROL1	16.26	19.82	20.56	<0.001	<0.001	0.23
P63104	14-3-3 protein zeta/delta	YWHAZ	20.30	19.83	19.30	0.04	<0.001	0.01
P07737	Profilin-1	PFN1	17.55	19.48	19.03	<0.001	<0.001	0.04
P13489	Ribonuclease inhibitor	RNH1	16.59	16.35	15.55	0.33	0.01	<0.001
P61769	Beta-2-microglobulin	B2M	15.16	17.60	18.28	<0.001	<0.001	0.14
P06703	Protein S100-A6	S100A6	20.45	18.23	17.69	<0.001	<0.001	0.16
P60660	Myosin light polypeptide 6	MYL6	18.34	16.83	16.31	<0.001	<0.001	0.12
P09228	Cystatin-SA	CST2	19.50	21.46	21.95	<0.001	<0.001	0.18
P07108	Acyl-CoA-binding protein	DBI	18.02	16.35	16.90	<0.001	0.02	0.02
P31949	Protein S100-A11	S100A11	14.61	18.09	17.65	<0.001	<0.001	0.36
O15145	Actin-related protein 2/3 complex subunit 3	ARPC3	10.11	14.35	13.49	<0.001	<0.001	0.09
Q12765	Secernin-1	SCRN1	13.41	14.76	14.30	<0.001	0.07	0.06
P15374	Ubiquitin carboxyl-terminal hydrolase isozyme L3	UCHL3	14.70	13.07	11.74	<0.001	0.01	0.02
P68402	Platelet-activating factor acetylhydrolase IB subunit beta	PAFAH1 B2	12.56	15.35	14.58	<0.001	<0.001	0.01

[a]Pairwise Welch's one-way analysis of variance (ANOVA)

Supplementary Table S3. Linear relationships between the baseline stratifying proteins and clinical signs.

Clinical sign	Uniprot	Full name	Symbol	Coefficients		P[a]	
				Intercept	protein expression (log_2)	unadj.	adj.
Schirmer's test as DV	P62258	14-3-3 protein epsilon	YWHAE	136.584	-6.764	<0.001	<0.001
	P13489	Ribonuclease inhibitor	RNH1	119.600	-6.454	<0.001	<0.001
	P11142	Heat shock cognate 71 kDa protein	HSPA8	132.799	-6.042	<0.001	<0.001
	P63104	14-3-3 protein zeta/delta	YWHAZ	131.371	-5.866	<0.001	<0.001
	P00390	Glutathione reductase, mitochondrial	GSR	105.298	-4.909	<0.001	<0.001
	P11021	78 kDa glucose-regulated protein	HSPA5	97.092	-4.802	<0.001	<0.001
	P60660	Myosin light polypeptide 6	MYL6	95.605	-4.702	<0.001	<0.001
	P02787	Serotransferrin	TF	88.786	-3.439	<0.001	<0.001
	P06703	Protein S100-A6	S100A6	68.254	-2.849	<0.001	<0.001
	P07108	Acyl-CoA-binding protein	DBI	53.620	-2.228	0.007	0.01
	P15374	Ubiquitin carboxyl-terminal hydrolase isozyme L3	UCHL3	38.406	-1.713	0.002	0.003
	P01036	Cystatin-S	CST4	-24.043	1.772	0.002	0.003
	P09228	Cystatin-SA	CST2	-34.424	2.365	<0.001	<0.001
	P61769	Beta-2-microglobulin	B2M	-25.904	2.396	<0.001	<0.001
	P01037	Cystatin-SN	CST1	-39.967	2.645	<0.001	<0.001
	Q99935	Proline-rich protein 1	PROL1	-35.713	2.683	<0.001	<0.001
FTBUT as DV	P11142	Heat shock cognate 71 kDa protein	HSPA8	69.072	-3.181	<0.001	0.010
	P63104	14-3-3 protein zeta/delta	YWHAZ	42.423	-1.769	0.002	0.018
	P00390	Glutathione reductase, mitochondrial	GSR	33.930	-1.445	0.001	0.016
	P02787	Serotransferrin	TF	27.844	-0.954	0.003	0.019

DV, dependent variable; FTBUT, fluorescein tear break-up time

[a]Mixed model regression

Supplementary methods

Denaturation, alkylation, reduction and tryptic digestion

Proteins were solubilized in 2% sodium dodecylsulfate (SDS) and reduced by 50 mM Tris-(2-carboxyethyl) phosphine (TCEP) for 60 min at +60°C. Samples were then transferred into 30 kDa molecular weight cut-off filters (Pall Corporation, Port Washington, New York, USA) and flushed two times with 8 M urea in 50 mM Tris-HCl (Merck KgaA, Darmstadt, Germany). Cysteine residues were blocked by iodoacetamide (IAA) at room temperature in the dark. Alkylation was terminated by centrifugation and the samples were flushed three times with urea solution. Three subsequent rinses with digestion buffer were performed prior to digestion with trypsin (Sciex, Framingham, MA, USA) for 16 h at +37°C at a trypsin-to-protein ratio of 1:25. Digests were dried in a speed vacuum concentrator and desalted with Pierce C18 tips (Thermo Fisher Scientific) according to manufacturer's instructions. After clean up the samples were once more vacuum dried and stored at -20°C until reconstituted to loading solution (5% ACN, 0.1% FA) at equal concentrations. Hyper reaction monitoring (HRM) peptide mix (Biognosys, Zurich, Switzerland) was added to each sample before sequential window acquisition of all theoretical fragment ion spectra (SWATH) analysis. All reagents were purchased from Sigma-Aldrich (St. Louis, MO, USA) unless otherwise stated. Two replicate MS analyses were performed from each sample.

NanoLC-TripleTOF

Digested peptides were analysed using Eksigent 425 NanoLC coupled with high speed TripleTOF 5600+ mass spectrometer (Ab Sciex, Concord, Canada). A capillary RP-LC column (cHiPLC ChromXP C18-CL, 3 µm particle size, 120 Å, 75 µm i.d × 15 cm, Eksigent Concord, Canada) was used for liquid chromatography separation of peptides. Samples were first loaded into trap column (cHiPLC ChromXP C18-CL, 3 µm particle size, 120 Å, 75 µm i.d × 5 mm) from autosampler and flushed for 10 min at 2 µl/min (2% ACN, 0.1% FA). The flush system was then switched to line with

analytical column. Tear samples were analysed with 120 min 6 step gradient using eluent A: 0.1% FA in 1% ACN and eluent B: 0.1% FA in ACN (eluent B from 5% to 7% over 2 min, 7% to 24% over 55 min, 24% to 40% over 29 min, 40% to 60% over 6 min, 60% to 90% over 2 min and kept at 90% for 15 min, 90% to 5% over 0.1 min and kept at 5% for 13 min) at 300 nl/min.

Key parameters for the mass spectrometer in SWATH ID library analysis were: ion spray voltage floating (ISVF) 2300 V, curtain gas (CUR) 30, interface heater temperature (IHT) +125°C, ion source gas 1 13, declustering potential (DP) 100 V. Library for SWATH analysis was created from the same samples by information dependent-acquisition (IDA) method and relative quantitation analysis was done by SWATH method. All methods were controlled by Analyst TF 1.5 software (Ab Sciex, USA). For IDA parameters, 0.25 s MS survey scan in the mass range 350-1250 mz followed by 60 MS/MS scans in the mass range of 100-1500 Da (total cycle time 3.302 s). Switching criteria were set to ions with mass to charge ratio (m/z) greater than 350 and smaller than 1250 (m/z), with charge state 2-5 and an abundance threshold of more than 120 counts. Exclusion of former target ions was set for 12 s. IDA rolling collision energy (CE) parameters script was set for automatically controlling CE. SWATH quantification analysis parameters were the same as for SWATH ID, with the following exceptions: cycle time 3.332 s and MS parameters set to 15 Da windows with 1 Da overlap between mass range 350-1250 Da followed by 40 MS/MS scans in the mass range of 100-1500 Da.
